# The Application of the Food Insulin Index in the Prevention and Management of Insulin Resistance and Diabetes: A Scoping Review

**DOI:** 10.3390/nu16050584

**Published:** 2024-02-21

**Authors:** Hildegard Strydom, Elizabeth Delport, Jane Muchiri, Zelda White

**Affiliations:** 1 Department of Human Nutrition, University of Pretoria, Pretoria 0084, South Africa; jane.muchiri@up.ac.za (J.M.); zelda.white@up.ac.za (Z.W.); 2 GI Foundation of South Africa, Nelspruit, Mbombela 1201, South Africa; liesbet@gifoundation.com

**Keywords:** food insulin index, insulin resistance, diabetes

## Abstract

The food insulin index (FII) is a novel algorithm used to determine insulin responses of carbohydrates, proteins, and fats. This scoping review aimed to provide an overview of all scientifically relevant information presented on the application of the FII in the prevention and management of insulin resistance and diabetes. The Arksey and O’Malley framework and the PRISMA Extension for Scoping Reviews 22-item checklist were used to ensure that all areas were covered in the scoping review. Our search identified 394 articles, of which 25 articles were included. Three main themes emerged from the included articles: 1. the association of FII with the development of metabolic syndrome, insulin resistance, and diabetes, 2. the comparison of FII with carbohydrate counting (CC) for the prediction of postprandial insulin response, and 3. the effect of metabolic status on the FII. Studies indicated that the FII can predict postprandial insulin response more accurately than CC, and that a high DII and DIL diet is associated with the development of metabolic syndrome, insulin resistance, and diabetes. The FII could be a valuable tool to use in the prevention and management of T1DM, insulin resistance, and T2DM, but more research is needed in this field.

## 1. Introduction

Insulin resistance is a disease classified by the resistance of cells to the function of insulin and may lead to the development of type 2 diabetes (T2DM) and heart disease [1,2]. In T2DM, the chronic hypersecretion of insulin as brought about by cellular insulin resistance may lead to beta-cell exhaustion and impaired insulin release, causing impaired glucose tolerance [3]. In type 1 diabetes (T1DM), the beta-cells are unable to produce insulin, and exogenous insulin needs to be administered via injections or an insulin pump, where the miscalculation of dosage and amounts and types of carbohydrates consumed often leads to hyper- or hypoglycaemia [3].

Carbohydrate counting (CC) has been used since the 1920s in dietary management of diabetes to determine carbohydrate amounts prescribed in diabetic diets [4,5]. CC rests on two main principles, namely, carbohydrates are the nutrients responsible for rises in blood glucose levels, and when any carbohydrates (fruit, pasta, rice, etc.) are eaten in certain amounts, blood glucose levels will rise in similar degrees (regardless of the type of carbohydrate eaten) [6]. However, this concept has been challenged by studies, showing that the equivalent amounts of carbohydrates from different carbohydrate sources elicit glycaemic responses that vary over a 4–5-fold range [7,8,9,10,11,12,13,14,15]. The glycaemic index (GI) of carbohydrates, a concept developed in 1980 by Jenkins and colleagues, has been researched by several studies, confirming that not all carbohydrates yield similar glycaemic or insulinemic responses [9,11,12,16,17,18,19,20,21,22]. While the GI proved a valuable tool to predict the postprandial effect of a single (carbohydrate) food on blood glucose levels, it cannot predict the effect a meal has on postprandial blood glucose levels. This led to the development and use of glycaemic load (GL) that takes both the amount of carbohydrate and the GI of the carbohydrate consumed in a meal or day into account [14,22,23]. A low GL diet has proven to decrease the risk of T2DM in many studies [17,22,24], and showed that the GL of a meal or food item will directly affect the amount of postprandial insulin secreted [9,13,15,25,26].

The food insulin index (FII) was first introduced by Holt and colleagues in 1997 when the postprandial insulin responses of thirty-eight isoenergetic foods (including carbohydrate-, protein-, and fat-rich food) were compared [27]. Other than CC, GI, or GL (where only carbohydrates are considered), the FII also takes the postprandial insulinemic effect of protein and fat into account [27]. Procedures for testing the FII are similar to the international standards for GI testing [28]. An insulin score (IS) is used to determine postprandial insulin responses to isoenergetic food (relative to white bread): a higher IS indicates an increased postprandial insulin response compared to a lower IS that indicates a reduced postprandial insulin response [27,29,30]. A glucose score (GS) for each test food can be calculated similar to the GI. The GS differs from GI by using 1000 kJ food portions compared to GI, where 50 g glycaemic carbohydrate portions are tested [7,12,27]. As the FII provides numeric values of IS and GS for each tested food [19], it can be used as a valuable tool during meal planning in the dietary treatment of insulin resistance to identify foods that will produce lower rises in postprandial blood insulin levels compared to other tested food (also consumed in 1000 kJ portions) and to determine portion size suggestions [27].

The dietary management of insulin resistance or diabetes should include limiting postprandial insulin levels. Therefore, there is a vital need to predict insulin secretion (to prevent or manage insulin resistance or T2DM) or insulin demand, in the case of T1DM. The aim of this scoping review was to produce an overview of all scientifically relevant information and research presented on the application of the FII in the management of insulin resistance and diabetes.

## 2. Materials and Methods

This review followed the Arksey and O’Malley (2005) [31] framework for scoping reviews. The methodology manual by the Joanna Briggs Institute for scoping reviews and recommendations by Levac et al. [32] were also consulted. The PRISMA Extension for Scoping Reviews (PRISMA-SCR) [33] 22-item checklist was used to ensure that all areas were covered in the scoping review. This approach includes the formulation of a research question, the selection of studies addressing the research question, and the summarisation and reporting of the results. The research question guiding the research was as follows: what is known about the FII in the prevention and management of insulin resistance and diabetes? The Population, Concept, Context (PCC) [34] eligibility criteria were as follows:

Population: all children, adolescents, and adults (from any ethnical group), healthy or diabetic, that received FII testing or dietary intervention involving the use of the FII.

Concept: dietary management and intervention specifically involving a calculated FII value of a meal or food item that was ingested, and postprandial glucose and insulin levels were monitored.

Context: data from all countries were included.

### 2.1. Search Strategy

Peer-reviewed, published literature as well as grey literature was searched with the assistance of a scoping review expert librarian. A comprehensive literature search was performed using the following electronic databases: Academic Search Complete, CINAHL, Cochrane CENTRAL, PUBMED, and SCOPUS. The terms “insulin index (II)”, “food insulin index (FII)”, and “dietary insulin index (DII)” were searched. Reference lists of relevant articles and peer-reviewed literature were hand-searched to identify relevant studies that were not listed in the electronic databases.

### 2.2. Eligibility Criteria

Original research articles were included if they reported on how the implementation of FII was linked to the prevention and management of either insulin resistance or diabetes. Articles were excluded if research was not performed on humans (animal studies) and if articles were published before 1997. Review papers, editorials, case studies, and opinion pieces were excluded.

### 2.3. Selection of Sources of Evidence

Records identified by the electronic databases were exported to Endnote 20, and imported into Rayyan (available at: https://rayyan.ai/ (accessed on 14 April 2023)), which was used as an electronic screening tool. Duplicates were removed. Two reviewers (H.S. and E.D.) independently conducted level 1 screening (screening of titles and abstracts) and level 2 screening (full-text screening) on all records for inclusion in the review. Discrepancies were discussed and resolved without the need to consult a third reviewer.

### 2.4. Charting the Data

A data extraction form was developed to chart data of all articles included in this study. The data that were extracted from each article included: author(s) and year of publication, city, and country where the study was performed, aim, study design, sample size and population, total number of participants, and main findings.

### 2.5. Summarizing and Reporting the Results

Data extracted from the included articles were thematically analysed and summarised. These themes were as follows: the association of FII with the development of metabolic syndrome, insulin resistance, and diabetes; the comparison of FII with CC for the prediction of postprandial insulin response; and the effect of metabolic status on the FII.

### 2.6. Quality Appraisal

The mixed methods appraisal tool (MMAT) version 2018 was used to evaluate the quality of included articles [35]. Two reviewers (H.S. and J.M.) independently appraised the methodological quality of the articles according to five categories of research: qualitative research, quantitative randomised controlled trials, quantitative non-randomised studies, quantitative descriptive studies, and mixed methods studies [35]. A score of ≤50% represented low-quality evidence, 50–75% represented average-quality evidence, and 76–100% presented high-quality evidence [35].

## 3. Results

### 3.1. Selection of the Included Articles

**Figure 1:**
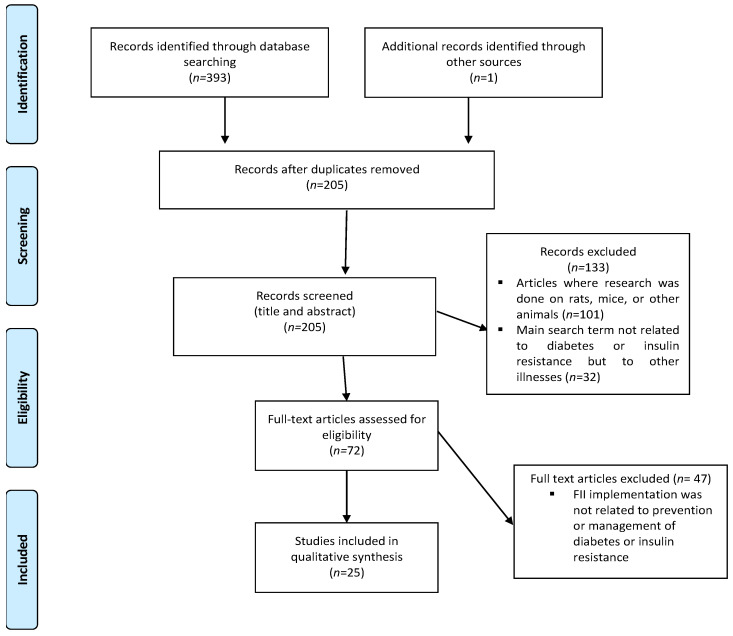
Preferred Reporting Items for Systematic Reviews and Meta-Analyses-Scoping Review (PRISMA-ScR) flow chart of literature search and selection of included articles.

Our search returned a total of 394 results, which consisted of 393 articles listed on electronic databases and 1 article from grey literature (Figure 1). After duplicates were removed, 205 articles remained, of which 134 articles were excluded because studies were not performed on humans (n = 101), and because FII was linked to other illnesses (not diabetes or insulin resistance) (n = 32). Full-text screening was performed on 72 articles of which 47 were excluded. Data extraction was performed on the remaining 25 articles [27,29,30,36,37,38,39,40,41,42,43,44,45,46,47,48,49,50,51,52,53,54,55,56,57].

### 3.2. Characteristics of the Included Articles

Of the twenty-five included articles, eleven were cross-sectional [29,36,37,38,39,48,50,51,54,55,56], six were crossover studies [30,41,43,46,47,52], four were randomised controlled trials (RCTs) [27,40,44], three were prospective studies [49,53,57], and one was a regression analysis [45]. Most of the studies were conducted in Australia (n = 11) and Iran (n = 10). Two of the articles were from Australia with co-authors from the US. The remainder of the studies were conducted in the UK (n = 2), Turkey (n = 2), Canada (n = 1), Italy (n = 1), and the USA (n = 1). Most of the articles (n = 13) were published between 2019 and 2023. The study populations of the articles included the following: four studies on type 2 diabetic individuals [36,37,38,39], seven studies on type 1 diabetic individuals [40,41,42,43,44,47,52], one study on obese adolescents with insulin resistance [46], eleven on healthy participants [27,29,45,48,49,51,53,54,55,56,57], one on both healthy and type 2 diabetic participants [30], and one study used healthy participants as well as participants with insulin resistance and T2DM [50]. Characteristics and findings of the included studies are summarised in Table 1.

### 3.3. Quality of Evidence

All 25 included articles were appraised with Mixed Methods Appraisal Tool (MMAT) version 2018 for methodological quality [35] (Table S1). One study scored 40%, representing low-quality evidence [52]. Five studies scored 50–75%. representing average-quality evidence [48,53,54,55,57], and 19 studies scored 76–100%, representing high-quality evidence [27,29,30,36,37,38,39,40,41,42,43,44,45,46,47,49,50,51,56].

### 3.4. Main Findings

**Figure 2:**
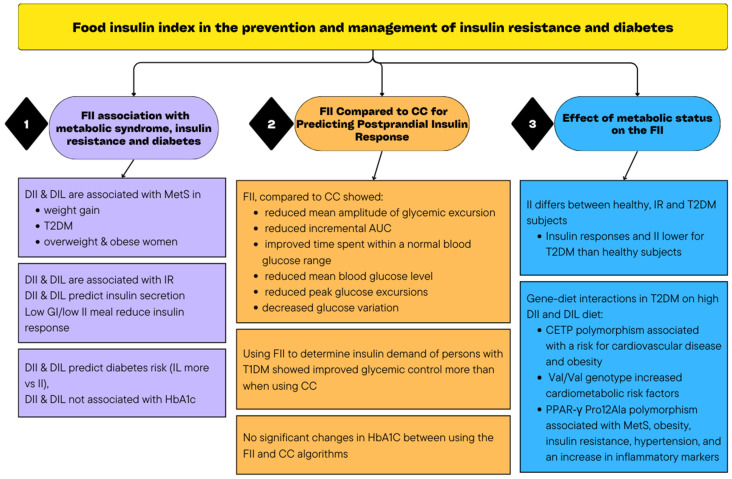
Summary of the findings of the scoping review.

All the articles that were included examined the effect of incorporating the FII to predict insulin response or how it is linked to diabetes or insulin resistance development or management. The findings are reported according to the identified main themes (Table 1). Figure 2 includes a visual summary of the findings of the scoping review.

#### 3.4.1. The Association of FII with the Development of Metabolic Syndrome, Insulin Resistance, and Diabetes

Ten studies examined the association of the FII with the development of insulin resistance, metabolic syndrome, and diabetes [39,46,48,49,51,53,54,55,56,57]. In most of these studies, the FII was used to determine insulin responses to meals by calculating dietary insulin index (DII) and dietary insulin load (DIL) [39,48,49,51,54,55,56].

Four studies in Iran examined whether an increased DII was associated with the development of metabolic syndrome [39,48,49,56]. One study found no significant association between DIL and DII and the risk of developing obesity and metabolic syndrome [48]. In contrast, a prospective study included a diet with a high DIL and DII, and it was significantly associated with higher risk for weight gain, and in women with weight stability, it was positively associated with the risk of developing metabolic syndrome. A higher risk of metabolic syndrome was seen with a diet high in DIL and DII in the group that gained weight and had low activity levels [49]. Sadeghi et al. studied healthy adults and found that higher DII and DIL were positively associated with the development of metabolic syndrome in women, while a moderate DIL was associated with increased risk of metabolic syndrome in men, whereas no significant association between high DII and metabolic syndrome was seen in men [56]. Anjom-Shoae et al., in a study conducted among people with T2DM, found that higher DIL and DII were positively associated with the risk of developing metabolic syndrome [39].

An association between DII and obesity was found in three studies, two in Iran [39,55] and one in Turkey [46]. Anjom-Shoae et al. [39] showed that higher DIL and DII were positively associated with abdominal obesity and that a higher DIl was associated with general obesity. Noori et al. also found a significant association between metabolically healthy overweight and obese women and DII and that inflammatory markers, e.g., IL-1β, may affect this association [55]. In addition, an association between a high II and increased appetite was reported by Caferoglu et al. [46]. In a randomised, single-blind, cross-over study, obese adolescents with insulin resistance received two test meals on different days [46]. The GI and macronutrient value of the meals were matched, but there was a two-fold difference in FII. Serum glucose, insulin, and C-peptide levels as well as appetite scores were recorded for early (0–30 min), late (45–240 min), and total (0–240 min) stages. Results showed a decrease of 25.8% and 27.5%, respectively, in the feeling of hunger in the late and total stages after the low-GI, low-II meal compared to the low-GI, high-II meal (*p* < 0.05). Postprandial insulin responses were lowered by 56.1% in the early stage, 34.6% in the late stage, and 35.6% in the total stage after a low-GI, low-II meal, compared to those of the low-GI, high-II meal (*p* < 0.05) [46].

In a 3-year prospective study in Iran on healthy adults, increased DIL was associated with an increased risk of insulin resistance, and increased DII had a borderline positive association with the development of insulin resistance [53].

Three studies examined whether DII and DIL were associated with an increased risk of developing diabetes [51,54,57]. C-peptide concentrations are seen to be a more valid indication of insulin resistance than insulin secretion [51]. Two studies in the USA examined how DII and DIL were associated with C-peptide concentrations [51,54]. Nimptsch et al. found no significant associations between DII and DIL and plasma HbA1C and C-peptide in healthy adults, indicating no significant association between DII and DIL and risk for diabetes [54]. In contrast, Lee et al. found that higher DII and DIL scores were associated with increased 24 h urinary C-peptide concentrations (and insulin secretion) in

Healthy men [51]. In a cohort study in Iran, to investigate the association of II, GI, IL, and GL per day with the risk of developing diabetes among healthy adults, it was found that although all four dietary scores were significantly associated with an increased risk of diabetes, IL and GL per day showed the strongest association (increasing the risk of diabetes by 70 and 84%, respectively). II was associated with a 33% increased risk for developing diabetes, and GI a 28% increased risk [57].

#### 3.4.2. The FII Compared to CC for Predicting Postprandial Insulin Response

Ten articles (nine Australian, one Turkish) examined whether using CC compared to the FII produced a better predicting value for postprandial insulin response [27,29,40,41,42,43,44,45,47,52].

In the first FII testing study, Holt et al. found that GS and IS were significantly correlated for most food; however, food high in protein as well as food high in both fat and refined carbohydrates (bakery products) elicited insulin responses that were much higher than their glucose responses [27]. High-protein food items often produced insulin secretions similar to the amounts of insulin secreted by carbohydrate-rich food and food with similar nutrient values elicited different insulin secretions. For example, food with similar carbohydrate contents produced different insulin responses. Therefore, the authors hypothesised that a meal’s insulinemic effect rather than the carbohydrate content should be used to predict postprandial response [27].

In order to predict the postprandial insulin demand using the FII, some of the studies [29,43,44,52] used the food insulin demand (FID) as prescribed by Bell et al. [43] (FID = FII × kJ per serving/1000) [43]. Prandial insulin dosage was determined using individualised insulin, i.e., FID ratio, which is similar to the insulin/carbohydrate ratio [29,43,44,52].

Two studies examined whether the FII could predict postprandial insulin response better than using CC, GL, or GI [29,45]. In a study by Bao et al. [29], GL, CC, and the FII were used to predict an insulin response of 13 isoenergetic meals consumed by healthy adults. The usage of the FID showed the highest correlation with the observed postprandial insulin responses (*p* = 0.0016). GL per meal also strongly correlated with the observed insulin response (*p* = 0.01) (although lesser than FII), while CC was not a significant predictor of the observed insulin responses (*p* = 0.064). The study also showed that two meals with similar observed insulin responses had markedly different carbohydrate contents (37 g and 63 g, respectively) [29]. Also, a meal with 40 g carbohydrates produced double the insulin response of a meal with a similar carbohydrate content of 37 g [29]. A study where mathematical algorithms were generated to improve the prediction of postprandial insulinemia found that GL per serving, GI, and glycaemic carbohydrate content were the strongest predictors of FII, but that glycaemic carbohydrate only accounted for 47% of the variation. Moreover, the FII could not be calculated by carbohydrate content alone, but all nutrients in the meal as well as their interactions are responsible for the postprandial insulin response [45].

Six studies that used both CC and the FII to determine the insulin demand of people with T1DM all found improved glycaemic control when using the FII (compared to using CC) [40,41,42,43,44,47]. In a study at the University of Sidney, Australia, among T1DM people on insulin pump therapy, the insulin requirements of six protein-containing single foods were determined once by using CC and once by using the FII to calculate an estimated food insulin demand [43]. Mean blood glucose levels at 180 min and mean change in blood glucose levels over 3 h were significantly lower when using the FII algorithm compared to those with CC (*p* = 0.003 and *p* = 0.001, respectively). In addition, the time to reach peak blood glucose levels were almost halved, demonstrating that the application of the FII (compared to CC) to single-protein-containing foods improved hyperglycaemia. The maximum amplitude of glycaemic excursion was significantly larger (4.4 ± 0.2 vs. 3.7 ± 0.2 mmol/L, *p* = 0.02) using the FII, and high rates of mild hypoglycaemia occurred during both treatments [43].

In a RCT, 26 people with T1DM on insulin pump therapy were assigned to either CC or FID counting, to calculate their prandial insulin demand over a 12-week period [44]. Results showed no significant changes in glycated haemoglobin (HbA1c) from baseline to 12 weeks in either group. The mean amplitude of glycaemic excursion, as well as the 120 min incremental area under the curve (AUC) following breakfast, was significantly reduced in the FID counters. The number of hyperglycaemic episodes, as well as time spent within normal blood glucose range, was similar for both groups, but the FID counters showed a reduced risk for hypoglycaemia [44]. Bao et al. [40] showed that when people with T1DM used either CC or FID counting to determine the prandial insulin dosage of breakfast meals, compared to carbohydrate counters, FII counters significantly improved the time spent within the normal blood glucose range, produced a significantly lower incremental AUC, reduced the time to reestablish the fasting blood glucose level, and caused a smaller peak glucose excursion. [40].

In a study in Turkey among adolescents with T1DM, participants consumed two meals with similar energy, macronutrient content, and FII, but with a two-fold difference in GI [47]. Insulin dosage for each meal was calculated once by using CC and once by using FID calculation. Results showed that for the high GI meal, compared to CC, the FII algorithm significantly decreased peak glucose excursion (−57%, *p* = 0.02) as well as the incremental AUC (−65%, *p* = 0.02), and the coefficient of glucose variation was decreased by 37% (*p* = 0.03). No significant difference was seen between using the CC and FII algorithms for the low GI meal, and no significant difference in the occurrence of hypoglycaemia was seen between any of the insulin-dosing algorithms in low- or high-GI meals [47].

Bell et al. used CC compared to the FII algorithm to estimate insulin dosage for six single foods in adults with T1DM and found, compared with CC, the FII algorithm significantly reduced mean blood glucose levels, produced a smaller mean change in blood glucose levels, and a smaller peak change in blood glucose excursion, without causing a significant risk of hypoglycaemia [41]. The same authors compared CC to the FII algorithm to predict insulin dosages for people with T1DM on insulin pump therapy over a 12-week period [42]. They found the FII counters showed a 43% reduction in hypoglycaemia at 12 weeks, while carbohydrate counters showed no change in hypoglycaemia. Both groups showed similar changes in HbA1c and postprandial glucose levels [42].

The only study that showed no significant difference in glucose excursions between CC and FII was a cross-over trial of children and adolescents with T1DM who consumed meals with similar carbohydrate content but with a high-protein or high-fat content using either CC or the FII algorithm to determine prandial insulin dose [52]. However, this study was the only one with a low-quality rating.

#### 3.4.3. The Effect of Metabolic Status on the FII

Two studies investigated whether II can differ among individuals based on their metabolic status [30,50]. In an Australian study, Bell et al. provided two diets matched for macronutrient and fibre content as well as GI, but with a two-fold difference in FII, to healthy and T2DM.adults. Postprandial plasma insulinemia and glycaemia was measured over 8 h. Results showed no difference in postprandial glycemia in either group or between the diets. However, the mean postprandial insulin response (over eight hours) was 53% lower in the healthy subjects on the low-FII diet compared to the high-FII diet, and 41% lower in the T2DM group on the low-FII diet compared to the high-FII diet [30]. In a cross-sectional study in Canada, GI and II were compared in healthy, hyperinsulinemic and T2DM subjects to investigate whether GI and II were dependent of the subjects’ metabolic status. The study found that GI values did not differ significantly between subject groups but that II values were higher in the T2DM group than in the healthy- or hyperinsulinemic groups and that II was inversely associated with insulin sensitivity [50].

Apart from differences in II due to individuals’ metabolic status, gene–diet interactions can further affect the metabolic status of individuals with T2DM, as seen in three Iranian studies [36,37,38]. One study found a significant interaction between cholesteryl ester transfer protein (CETP) polymorphism and DIL and DII and their effect on obesity indices (waist circumference and BMI), lipid profiles (triglycerides, high-density lipoproteins (HDL) and low-density lipoproteins (LDL) to high density lipoprotein ratio, inflammatory markers (interleukin-18, C-reactive protein, and prostaglandin F2-α), and antioxidant markers (total antioxidant capacity and superoxide dis-mutases)) in patients with T2DM. The study suggested that CETP polymorphism may be associated with a risk for cardiovascular disease in patients following an increased DII and DIL diet [36]. In another study, it was found that individuals with T2DM and the Val/Val genotype of the BDNF (brain-derived neurotropic factor) gene were more likely to be at risk of cardiovascular disease, compared to subjects carrying Met-alleles. Individuals with the Met/Val or Met/Met genotypes had lower BMI, serum leptin, and total cholesterol levels, even when they consumed diets higher in the DIL index compared to individuals with the Val/Val genotype. The highest quartile of DIL, showed an increase in waist circumference and LDL/HDL for the Val/Val homozygotes compared with Met-allele carriers. The study recommended that T2DM people with the Val/Val genotype should especially follow a low GI, daily low GL, low DII and DIL diet for protection from reduced insulin sensitivity and cardiometabolic risk factors [37]. A cross-sectional study in Iran among patients with T2DM showed that PPAR-γ Pro12Ala polymorphism was able to increase the effect of DII and DIL and that it is associated with metabolic syndrome, obesity, insulin resistance, hypertension, and an increase in inflammatory markers [38].

## 4. Discussion

This scoping review was conducted to summarise the evidence available on how the FII can be implemented in the dietary prevention and management of diabetes and insulin resistance. We identified 25 studies published between 1997 and 2023 addressing this topic with three main research themes.

The World Health Organization (WHO) has reported a continued rise in numbers, with a reported 1.5 million deaths caused directly by diabetes in 2019. These alarming figures emphasise the importance of evidence-based intervention in not only the management but also the prevention of insulin resistance and T2DM. As insulin resistance is caused by chronic high concentrations of insulin, intervention should focus on limiting postprandial insulin secretion. Our findings indicate that the majority of the included studies showed an association between an increased DII and DIL and the development of metabolic syndrome (including insulin resistance) as well as weight gain [39,49,53,56], and the development of T2DM [51,57]. Therefore, the FII could potentially be used as a guide to choose food with a lower DII and DIL to lower the risk for the development of insulin resistance and T2DM. More research and guidelines are needed on how the FII can be practically implemented to achieve this.

When the ability of FII to predict postprandial insulin response was compared to CC, FII showed a significant correlation to observed insulin responses while CC was found not to be a significant predictor of observed insulin responses [29]. In addition, studies showed that the FII, compared to CC, reduced the mean amplitude of glycaemic excursion [41,43,44], reduced the incremental AUC [40,44,47], improved time spent within a normal blood glucose range [40], reduced mean blood glucose levels [41], reduced peak glucose excursions [40,41,47], and decreased glucose variation, These findings indicate that, if high GI carbohydrates are consumed as part of a mixed meal, FII should preferably be used instead of CC for better control of blood glucose levels. However, for a low GI meal, studies showed no significant differences between using CC and the FII algorithms, indicating that if the carbohydrate content of a mixed meal has a low GI value, CC could be used and should be effective in predicting the insulin demand of the meal [47]. Although no significant changes were seen in HbA1C levels between using the FII and CC algorithms [42,44], a reduced risk of hypoglycaemia was observed [42,44]. Contrary to that, one study found that the peak glucose excursion was significantly larger for the FII group compared to that of the CC group, and high incidences of mild hypoglycaemia were found in both the FII and CC groups [44]. In contrast, another study found no difference in glucose excursion between FII and CC groups [49], even though the majority of the included studies found the FII to be more beneficial in predicting postprandial insulin levels and/or improving glycemia and insulinemia. However, CC is still considered the gold standard in the calculation of insulin dosages and is compulsory to initiate insulin pump therapy [43,44].

Results from FII testing showed that foods containing similar carbohydrate amounts produced very different insulin scores [27,43]. Also, foods with a high-carbohydrate content can elicit low levels of insulin secretion (this could be due to the difference in their GI values and GL values per serving), and that ingestion of high-protein and high-fat food items can induce insulin responses similar to those of a high-carbohydrate meal [27,29,43]. These results challenge the validity of CC, as CC treats all carbohydrates consumed in similar amounts to elicit equal insulin responses and disregards their GI values and GL values per serving/meal, as well as the effects that protein and fat may have on postprandial insulin responses. As studies have shown that in order to rank food according to their insulin demand, the knowledge of glycaemic response together with nutrient composition should be considered [27,45], and using the FID to calculate insulin demand proved to be a more reliable option [29,43,44], since it takes into account the GI values of the carbohydrate foods, the mean GI value and the GL value of the carbohydrate component of the meal, and the protein and fat content of the meal. However, the FII needs to be expanded to include more foods. Our study highlights the need for studies focusing on evidence using the FII as a tool to calculate insulin demand (and determine exogenous insulin dosage) of mixed meals, especially for individuals with T1DM. This should assist greatly in the prevention of hypoglycaemia and hyperglycaemia at different times, the reduction and stabilisation of the insulin dosage taken, and the stabilisation of blood glucose levels.

This scoping review identified two other factors that need consideration when implementing the II, namely, metabolic status and gene interactions. Firstly, results showed that the postprandial effect of the II could differ depending on metabolic status. Subjects with T2DM showed lower insulin responses and lower subsequent II values than healthy subjects or subjects with insulin resistance [30,50]. This contradicts studies on GI, which do not differ according to metabolic status [30,50], suggesting a need to adjust II tables to be specific for metabolic status such as healthy and T1DM or T2DM. Secondly, the presence of certain genes was associated with an increased risk for heart disease and metabolic syndrome in patients following an increased DII and DIL [36,37,38]. This indicates that gene testing and a patient’s gene profile could be considered together with their diet, but more research is needed before recommendations can be made for practise.

### Strengths and Limitations

The notable strength of this scoping review is the robust methodology used whose implementation was ensured in each step using standards and guidelines from several recognised organisations. The inclusion of grey literature as part of the search strategy also reduced publication bias, by increasing the comprehensiveness of the available evidence reviewed for inclusion in this scoping review.

Included studies were evaluated for their quality of evidence based on their methodological quality using the MMAT, with majority (76%) of included studies representing high-quality evidence. In addition, this scoping review provided a summary of the evidence supporting the role of FII in the prevention and management of insulin resistance and diabetes, thereby identifying gaps and future research needs of selected themes to add to the existing body of knowledge, with the aim of formulating practise guidelines for the use of the FII in diabetes management.

A limitation of this review is that because most articles included in this study are limited to two countries (Australia or Iran), it may limit the representation of results to a wider population. However, this highlights a gap in research that could urge researchers from other countries to contribute research in this field as well.

## 5. Conclusions

This scoping review indicates that the FII can be used to predict postprandial insulin response and determine insulin dosage for individuals with T1DM more accurately than CC. Increased DII and DIL are linked to the development of insulin resistance and T2DM, and more research is needed to determine how the FII can be implemented practically. Factors such as metabolic status and the presence of specific genes should also be considered in the treatment of metabolic syndrome and diabetes. The FII could ultimately be a valuable tool for use in the dietary prevention and management of T1DM, insulin resistance, and T2DM, but more research is needed in this field.

## Figures and Tables

**Table 1:** Characteristics and findings of included studies per theme.

Author	City, Country	Aim	Study Design	Sample Size and Population	Main Findings
Theme 1: The association of FII with the development of metabolic syndrome, insulin resistance and diabetes					
Mirmiran et al., 2016 [53]	Tehran, Iran	To investigate the relationship between DII and DIL and the risk of development of insulin resistance in adults.	PS	927 adults Mean age: 40.7 ± 12.4 year BMI: 27.3 ± 4.9 kg/m^2^	DII showed a borderline positive association with insulin resistance. Increased DIL was associated with an increased risk of insulin resistance. Food with high FII can put individuals at higher risk of developing insulin resistance.
Sadeghi et al., 2019 [56]	Yazd, Iran	To determine the association between DII and DIL and the development of MetS.	CS	5954 adults 35–70 years	In men: Moderate DIL was associated with increased odds of metabolic syndrome. No significant association was seen for DII. In women: DIL was significantly associated with increased odds of developing MetS. A higher score of DII was associated with 41% greater odds of developing MetS.
Ghorbaninejad et al., 2021 [48]	Tehran, Iran	To examine the association between the insulinemic potential of a diet and MetS and obesity amongst Iranian adults.	CS	850 healthy adults, 20–59 years	No significant association between DIL and DII and the risk of MetS and obesity amongst the Iranian population was found.
Teymoori et al., 2021 [57]	Tehran, Iran	To investigate the association between II, IL, GI and GL and the risk of developing diabetes among the Tehranian adults	Prospective study	1149 healthy adults	The IL and GL (more than the II and GI) can strongly predict the risk of developing diabetes.
Khoshnoudi-Rad et al., 2022 [49]	Tehran, Iran	To investigate: 1. The association between DII and DIL and the development of MetS. 2. The extent to which sex and lifestyle (physical activity, smoking status, and weight change) influence the relationship between DII, DIL, and MetS risk.	Prospective study	1915 adults	DII and DIL were associated with weight gain, but not with the development of MetS. DII higher or lower than the median intake, was positively associated with the risk of MetS in the weight gain group. A higher risk of metabolic syndrome with a diet high in DII and DIL, weight gain and low levels of physical activity. In women with stable weight, a higher dietary DII and DIL increased the risk of MetS.
Noori et al., 2022 [55]	Tehran, Iran	To investigate the relationship of inflammatory factors (IL-1αand TGF-α) with DII and DIL on healthy and unhealthy metabolic phenotypes in obese and overweight adult women.	CS	Healthy adults (women) 18–48 years	Significant associations were shown between the group of metabolically healthy overweight and obese women and DII and DIL intake. IL-1β might play a role in this association.
Anjom-Shoae et al., 2023 [39]	Kermanshah, Iran	To evaluate the link between the DII and DIL and cardiometabolic risk factors in patients with T2DM mellitus.	CS	827 T2DM adults 40–70 years	Higher DII and DIL were shown to be positively associated with higher odds of MetS and abdominal obesity among patients with T2DM. A significant association was also shown between DIL and general obesity.
Nimptsch et al., 2011 [54]	Boston, USA	To investigate the average dietary II and IL in relation to biomarkers of glycaemic control, plasma lipids, and inflammation markers.	CS	4002 healthy adults	Participants in the highest quintile of II had 26% higher triglyceride concentrations than participants in the lowest quintile of II. This association was most common in obese participants. DII and DIL were inversely associated with HDL cholesterol in obese participants. No significant association between DII and DIL and plasma C-peptide, HbA1c, LDL cholesterol, CRP, or IL-6.
Lee et al., 2020 [51]	Boston, USA	To assess whether DII and empirical dietary index for hyperinsulinemia are predictive of insulin secretion.	CS	293 healthy adult men Younger than 70 years	DII, DIL and EDIH were found to be predictive of insulin secretion assessed by 24-h urinary C-peptide.
Caferoglu et al., 2019 [46]	Kayseri, Turkey	Comparing postprandial metabolic responses and appetite after ingestion of two meals with similar macronutrient content and GI with either high or low II in obese adolescents with insulin resistance.	RCOT	15 obese adolescents with insulin resistance 12–18 years Weight ≥ 95th	Postprandial insulin responses and feelings of hunger were significantly reduced with the low GI/low II meal compared to the low GI/high II meal.
Theme 2: The FII Compared to CC for Predicting Postprandial Insulin Response					
Holt et al., 1997 [27]	Sydney, Australia	To compare the postprandial insulin responses of isoenergetic food.	RCT	41 healthy adults BMI: 22.7 ± 0.04	Significant differences in insulin scores were found within food with similar carbohydrate content and GI. Some fat- and protein-rich food induced similar insulin responses to carbohydrate-rich food. Fibre content did not predict insulin response (similar responses between white and whole wheat counterparts).
Bao et al., 2009 [29]	Sidney, Australia	To determine whether using the FII was able to predict insulin responses to mixed meals compared to using GL or CC.	CS	21 healthy adults Mean age: 24 ± 2.5 years BMI: 19–25	The degree of postprandial hyperinsulinemia elicited by realistic mixed meals was best predicted by using the FII followed by GL and fat content, although unsaturated fats have a more favourable response than saturated fats. Carbohydrate, fibre, and protein content were found to be relatively poor predictors of the overall insulin response.
Bao et al., 2011 [40]	Sidney, Australia	To determine whether using the FII algorithm to determine mealtime insulin demand will reduce the severity of blood glucose fluctuations (without causing hypoglycaemia) more than using CC.	RCT	28 T1DM adults 18–70 years Diabetic diagnosis ≥1 year On insulin pump therapy	Compared with CC, the FII algorithm significantly decreased peak glucose excursion, glucose incremental area under the curve over 3 h (−52%, *p* = 0.013) and improved the percentage of time within the normal blood glucose range (4–10 mmol/L) (31%, *p* = 0.001). No significant difference in the occurrence of hypoglycaemia was seen between the two algorithms.
Bell et al., 2013 [41]	Sydney, Australia	To compare CC and the FII algorithm for estimating insulin dosage in adults with T1DM consuming six different single food.	TBR within-subject cross-over-controlled trial	11 T1DM adults	Compared with CC, the FII algorithm significantly reduced mean blood glucose levels, produced a smaller mean change in blood glucose levels and a smaller peak change in blood glucose excursion, without causing a significant risk for hypoglycaemia.
Bell et al., 2014 [43]	Sidney, Australia	To compare postprandial glycaemic responses in adults with T1DM using CC and the FII algorithm to estimate the insulin dosage for protein-containing foods.	TBR within-subject cross-over design	11 T1DM adults 18–70 years diabetic diagnosis ≥1 year On insulin pump therapy	For single, protein-containing foods, the application of FII (compared to CC) reduced the mean blood glucose level by an average of ~12% and halved the time to reach peak blood glucose over a 3-H testing period. FII caused higher mean amplitudes of glycaemic excursion compared with CC. FII and CC were both associated with relatively high rates of mild hypoglycaemia.
Bell et al., 2014 [42]	Sydney, Australia	To compare if changes in HbA1c levels occurred when using CC versus the FII algorithm for estimating insulin dosages over 12 weeks in adults with type I diabetes using an insulin pump.	RCT	26 adults with T1DM	The FII counters showed a 43% reduction in hypoglycaemia at 12 weeks, while CC counters showed no change in hypoglycaemia. Both groups showed similar changes in HbA1c and postprandial glucose levels.
Bell et al., 2016 [44]	Sidney, Australia and Boston, US	Comparing postprandial glycemia results over 12 weeks of using CC versus using the FII when estimating insulin dosage.	RCT	26 T1DM adults 18–70 years old Diabetic diagnosis ≥1 year	No significant changes in HbA1c over 12 weeks in both groups. The incremental area under the curve following breakfast declined significantly among the FII demand counters with no change in the CC group. Significant reduction in the mean amplitude of the glycaemic excursion among the FII demand counters. Only FII counters and not CC counters experienced a trend of reduced hypoglycaemia.
Bell et al., 2016 [45]	Sydney, Australia	The aim was to generate mathematical algorithms to improve the prediction of postprandial insulin secretion for foods of known nutrient composition, GI and GL.	Regression analysis	-	GL, GI and available carbohydrate content were the strongest predictors of FII. Macronutrient composition alone cannot predict postprandial insulin secretion and that at least one measure of glycaemic impact (GI, GL, GS) is required.
Lopez et al., 2018 [52]	New-castle, Australia	To compare the impact of using Pankowska Equation and FII algorithms to CC on postprandial glucose excursions following a high fat and a high protein meal.	RCOT	33 T1DM children and adolescents 7–14 years old Diabetic diagnosis ≥1 year Insulin pump therapy ≥6 months	The Pankowska Equation reduced postprandial hyperglycaemia, but increased hypoglycaemia. No significant differences when CC was compared to the FII.
Erdal et al., 2021 [47]	Kayseri, Turkey	Comparing the differences in the postprandial glycaemia when using CC and the food insulin index algorithms following the consumption of protein-rich, high-fat meals with different glycaemic index values.	RSBCT	15 T1DM adolescents 14–18 years	In the high GI meal: compared with CC, the FII algorithm significantly decreased peak glucose excursion (−57%, *p* = 0.02), incremental area under the curve (−65%, *p* = 0.02) and coefficient variation of blood glucose (−37%, *p* = 0.03). No difference between the two algorithms in the low-GI meal. No significant difference in the occurrence of hypoglycaemia between the two algorithms.
Theme 3: The effect of metabolic status on the FII					
Lan-Pidhainy et al., 2011 [50]	Toronto, Canada	To investigate if GI and II values of carbohydrate-rich foods are similar in healthy, hyperinsulinemic and T2DM subjects, and whether metabolic status (insulin sensitivity, b-cell function, fasting-and postprandial-glucose, hepatic insulin extraction and plasma GLP-1 response) of the subjects influence the GI and II values.	CS	31 Healthy, hyperinsulinemic, and T2DM adults	GI was not significantly different between the three groups; therefore, GI is not influenced by metabolic status. II was different between the three groups and may depend upon the glycaemic control, insulin sensitivity and hepatic insulin extraction of the subjects, therefore II may be influenced by metabolic status.
Bell et al., 2015 [30]	Sydney, Australia	To compare postprandial glucose and insulin responses to 3 consecutive meals of 2 nutrient-matched diets predicted to have either high or low insulin demand in healthy controls and participants with T2DM.	RCT	20 Healthy adults and T2DM adults	No differences in glycaemic responses between the 2 diets (similar GI’s) in either group. Compared with the high-FII diet, mean postprandial insulin response over 8 h was 53% lower with the low-FII diet in healthy subjects and 41% lower in subjects with T2DM.
Abaj et al., 2021 [36]	Tehran, Iran	To examine the interaction of CETP TaqB1 polymorphism with DII and DIL in altering cardiovascular risk factors among T2DM.	CS	220 T2DM adults 134 female 86 male Mean age: 52.2 years	A significant interaction of CETP rs708272 polymorphism with DIL and DII in T2DM patients on: obesity indices (waist circumference and BMI) lipid profiles (TG, HDL, LDL/HDL) inflammatory markers (IL-18, CRP, and PGF2α), antioxidant markers (TAC and SOD).
Abaj et al., 2022 [37]	Tehran, Iran	To investigate whether an interaction between BDNFVal66Met polymorphisms and DII and DIL can affect cardiometabolic markers among diabetic patients.	CS	667 T2DM adults 407 female 260 male (35–65 years)	Individuals with the Met/Val or Met/Met genotype had lower BMI, leptin, and total cholesterol when they consumed diets higher in the DIL and DII compared to individuals with the Val/Val genotype. The highest quartile of DIL, showed an increase in waist circumference and LDL/HDL for Val/Val homozygotes compared with Met-allele carriers. BDNF Val66Met variants may interact with DIL and DII and cause the development of cardiometabolic risk factors. Diabetic patients with Met alleles can regulate dietary intakes to regulate their cardio-metabolic markers.
Abaj et al., 2022 [38]	Tehran, Iran	To examine the interaction of Pro12Ala polymorphism with DII and DIL in altering cardiovascular risk factors among T2DM.	CS	393 T2DM adults	DII and DIL interact with the PPAR-γ Pro12Ala polymorphism and are associated with metabolic syndrome, obesity, insulin resistance, hypertension, and an increase in inflammatory markers in patients with T2DM. No association between DIL and lipid profiles was found.

DII: dietary insulin index; DIL: dietary insulin load; T2DM: type 2 diabetes; CS: cross-sectional; BMI: body mass index; TG: triglycerides, HDL: high density lipoproteins, LDL: low density lipoproteins; IL: interleukin, CRP: C-Reactive protein; PGF: Prostaglandin F, TAC: total antioxidant capacity; SOD: superoxide dismutase; BDNF: brain-derived neurotrophic factor; T1DM: type 1 diabetes, CC: carbohydrate counting; FII: food insulin index; TBR: triple-blinded randomised; RCT: randomised control trial; HbA1C: haemoglobin A1C; GL: glycaemic load; GI: glycaemic index; RSBCT: randomised, single-blind crossover trial; PS: prospective study; MetS: metabolic syndrome; TGF: transforming growth factor; II: insulin index, IL: insulin load; and RCOT: randomised cross-over trail.

## Data Availability

Data is contained within the article or Supplementary Material.

## Supplementary Materials

The following supporting information can be downloaded at: https://www.mdpi.com/article/10.3390/nu16050584/s1, Table S1. Quality appraisal of included studies.
