# Supplementary material for: The Application of the Food Insulin Index in the Prevention and Management of Insulin Resistance and Diabetes: A Scoping Review
Source: Nutrients. 2024 Feb 21;16(5):584. doi: 10.3390/nu16050584 (PMC10934417; doi:10.3390/nu16050584)
Supplement: Supplementary file 1 [file nutrients-16-00584-s001.zip › nutrients-2752460-supplementary.pdf]

Supplementary Materials

The Application of the Food Insulin Index in the Prevention and Management of Insulin Resistance and Diabetes: A Scoping Review

Hildegard Strydom [1,*], Elizabeth Delport [2], Jane Muchiri [1] and Zelda White [1]

Table S1. Quality appraisal of included articles.

			SCREENING QUESTIONS		1. QUALITATIVE STUDIES					Score (%)
RefID	*First author*	Year	S1. Are there clear research questions?	S2. Do the collected data allow to address the research questions?	1.1. Is the qualitative approach appropriate to answer the research question?	1.2. Are the qualitative data collection methods adequate to address the research question?	1.3. Are the findings adequately derived from the data?	1.4. Is the interpretation of results sufficiently substantiated by data?	1.5. Is there coherence between qualitative data sources, collection, analysis and interpretation?	
-	-	-	-	-	-	-	-	-	-	-
					2. QUANTITATIVE RANDOMIZED CONTROLLED TRIALS					
					2.1. Is randomization appropriately performed?	2.2. Are the groups comparable at baseline?	2.3. Are there complete outcome data?	2.4. Are outcome assessors blinded to the intervention provided?	2.5 Did the participants adhere to the assigned intervention?	
43	*Bell*	2014	YES	YES	YES	YES	YES	YES	YES	100
44	*Bell*	2016	YES	YES	YES	YES	YES	YES	YES	100
40	*Bao*	2011	YES	YES	YES	YES	YES	CAN'T TELL	YES	80
47	*Erdal*	2021	YES	YES	YES	YES	YES	CAN'T TELL	YES	80
30	*Bell*	2015	YES	YES	YES	YES	YES	YES	YES	100
52	*Lopez*	2018	YES	YES	YES	YES	NO	CAN'T TELL	CAN'T TELL	40
46	*Caferoglu*	2019	YES	YES	YES	YES	YES	NO	YES	80
41	*Bell*	2013	YES	YES	YES	YES	YES	YES	YES	100
42	*Bell*	2014	YES	YES	YES	YES	YES	YES	YES	100
					3. QUANTITATIVE NON-RANDOMIZED TRAILS					
					3.1. Are the participants representative of the target population?	3.2. Are measurements appropriate regarding both the outcome and intervention (or exposure)?	3.3. Are there complete outcome data?	3.4. Are the confounders accounted for in the design and analysis?	3.5. During the study period, is the intervention administered (or exposure occurred) as intended?	
36	*Abaj*	2021	YES	YES	NO	YES	YES	YES	YES	80
37	*Abaj*	2022	YES	YES	YES	YES	YES	YES	YES	100
29	*Bao*	2009	YES	YES	YES	YES	YES	YES	YES	100
38	*Abaj*	2022	YES	YES	YES	YES	YES	YES	YES	100

49	*Khoshnoudi-Rad*	2022	YES	YES	YES	YES	YES	YES	YES	100
53	*Mirmiran*	2016	YES	YES	NO	YES	NO	YES	YES	60
56	*Sadeghi*	2019	YES	YES	YES	YES	CAN'T TELL	YES	YES	80
39	*Anjom-Shoae*	2023	YES	YES	YES	YES	YES	CAN'T TELL	YES	80
48	*Ghorbaninerjad*	2021	YES	YES	YES	YES	NO	NO	YES	60
51	*Lee*	2020	YES	YES	YES	YES	YES	CAN'T TELL	YES	80
55	*Noori*	2022	YES	YES	YES	YES	NO	NO	YES	60
57	*Teymoori*		YES	YES	YES	YES	NO	NO	YES	60
54	*Nimpsch*	2011	YES	YES	CAN'T TELL	YES	NO	YES	YES	60
50	*Lan-Pidhainy*	2011	YES	YES	YES	YES	YES	YES	YES	100
27	*Holt*	1997	YES	YES	YES	YES	YES	CAN'T TELL	YES	80
							**4. QUANTITATIVE DESCRIPTIVE**			
					**4.1. Is the sampling strategy relevant to address the research question?**	**4.2. Is the sample representative of the target population?**	**4.3. Are the measurements appropriate?**	**4.4. Is the risk of nonresponse bias low?**	**4.5. Is the statistical analysis appropriate to answer the research question?**	
45	*Bell*	2016	YES	YES	YES	YES	YES	N/A	YES	100
							**5. MIXED METHODS**			
					5.1. Is there an adequate rationale for using a mixed methods design to address the research question?	5.2. Are the different components of the study effectively integrated to answer the research question?	5.3. Are the outputs of the integration of qualitative and quantitative components adequately interpreted?	5.4. Are divergences and inconsistencies between quantitative and qualitative results adequately addressed?	5.5. Do the different components of the study adhere to the quality criteria of each tradition of the methods involved?	
-	-	-	-	-	-	-	-	-	-	-
